# High Systemic Immune-Inflammation Index and Body Mass Index Are Independent Risk Factors of the Thoracic Ossification of the Ligamentum Flavum

**DOI:** 10.1155/2022/4300894

**Published:** 2022-08-13

**Authors:** Yongzhao Zhao, Qian Xiang, Jialiang Lin, Shuai Jiang, Weishi Li

**Affiliations:** ^1^Department of Orthopaedics, Peking University Third Hospital, Beijing, China; ^2^Beijing Key Laboratory of Spinal Disease Research, Beijing, China; ^3^Engineering Research Center of Bone and Joint Precision Medicine, Ministry of Education, Beijing, China

## Abstract

**Background:**

Inflammation has been considered to play an important role in the pathogenesis of the thoracic ossification of the ligamentum flavum (OLF). However, the inflammation-related risk factors of thoracic OLF have not been fully investigated to date.

**Methods:**

A total of 95 patients (48 in the OLF group and 47 in the control group) were included in this retrospective study to explore the independent risk factors of thoracic OLF. The following demographic and clinical variables were compared between the two groups: gender, age, body mass index (BMI), coexistence of hypertension or diabetes, and inflammation-related variables. Multivariate logistic regression analysis was utilized to determine the independent risk factors.

**Results:**

High systemic immune-inflammation index (SII) (≥621) (odds ratio [OR] = 12.16, 95% confidence interval [CI] = 2.95–50.17, *p* < 0.01) and BMI (≥25 kg/m^2^) (OR = 9.17, 95%CI = 3.22–26.08, *p* < 0.01) were independent risk factors of thoracic OLF. SII (*R* = 0.38, *p* < 0.01) and BMI (*R* = 0.46, *p* < 0.01) were positively associated with OLF score.

**Conclusion:**

High SII and BMI were the independent risk factors of thoracic OLF. Multicenter prospective studies with a large population should be conducted in the future to verify our findings.

## 1. Background

The thoracic ossification of the ligamentum flavum (OLF) has become the leading cause of thoracic spinal stenosis, which can compress the spinal cord and lead to thoracic myelopathy [1, 2]. Patients with OLF-induced thoracic spinal stenosis often ask for help with the complaints of weakness and the paraesthesia of the lower limbs, paraesthesia of the trunk, and loss of bladder or bowel control [2]. A position for the conservative treatment for thoracic OLF does not exist because of unsatisfactory results [2]. Posterior laminectomy is the standard treatment for thoracic spinal stenosis caused by OLF; however, its surgical process is challenging and associated with the high risk of perioperative complications [3, 4]. Therefore, seeking relevant biomarkers for thoracic OLF is necessary to improve the mechanistic investigation and therapy of this disease.

Thoracic OLF has been considered as a multifactorial disease, and several pathogenic factors, including genetic inheritance, metabolic disorders, inflammation, and mechanical stress, contribute to its onset and development [5–7]. Several studies have been conducted to identify the risk factors of thoracic OLF to further explore its underlying mechanisms [8–12]. Zhang et al. found that age and body mass index (BMI) were independent risk factors of thoracic OLF [8]. Tang et al. reported that new OLF was associated with high BMI and that the size progression of OLF was related to high BMI and smoking [9]. Liang et al. found that large thoracic kyphosis, lumbar lordosis, and sacral slope were associated with thoracic OLF; these associations indicated that mechanical stress might contribute to the development of OLF [10]. Inflammation is another important pathogenic factor of OLF, and several inflammatory cytokines, such as IL-6 and TNF-*α*, promote the onset and development of OLF [11, 12]. However, no inflammation-related risk factor of OLF has been identified to date.

Systemic inflammatory responses have been demonstrated to be associated with several nonimmunological diseases, including cancer [13, 14], intervertebral disc degeneration [15], and ectopic ossification [16]. Several clinical models consisting of leukocytes from the circulatory system were constructed for the further investigation of the role of systemic inflammatory responses in diseases and have been proven to have satisfactory diagnostic or prognostic value in human diseases [17–20]. Neutrophil-to-lymphocyte ratio (NLR), platelet-to-lymphocyte ratio (PLR), monocyte-to-lymphocyte ratio (MLR), and systemic immune-inflammation index (SII) are the most commonly used inflammatory biomarkers among existing models and have been verified to play an unfavorable role in the prognosis of several human diseases, especially cancers [21–23]. However, the relationship between inflammatory biomarkers and the development of OLF has not been investigated until now.

In this study, we compared the demographics and clinical variables of patients with thoracic OLF with those of patients without thoracic OLF to determine the inflammation-related risk factors of OLF.

## 2. Materials and Methods

### 2.1. General Information

**Figure 1:**
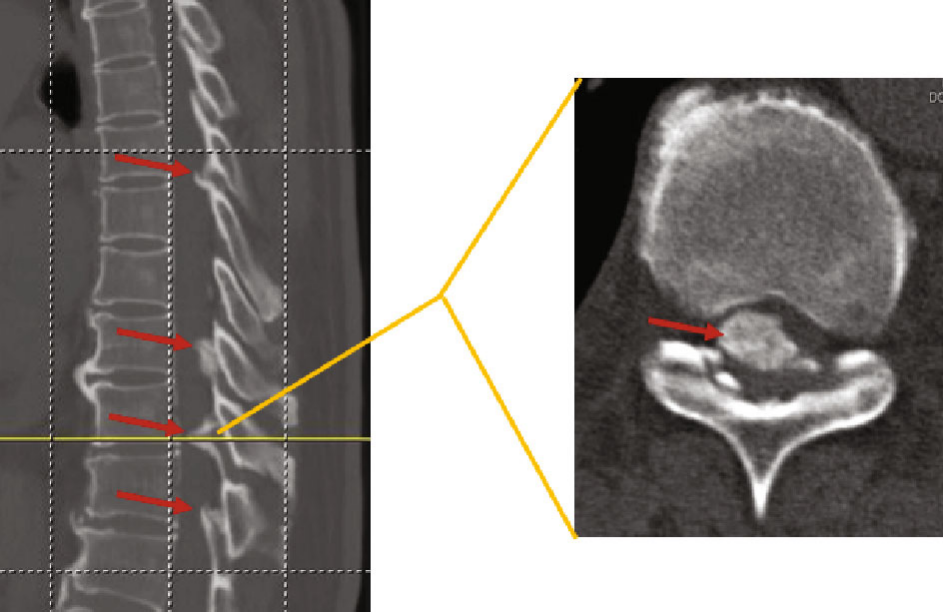
Ossified ligamentum flavum (red arrow) in thoracic spine.

This study was approved by the Ethics Committee of Peking University Third Hospital. Informed consent was not needed given the retrospective design of this study. Patients receiving posterior laminectomy for thoracic OLF from March 2021 to October 2021 at our hospital were included in the OLF group. Inflammation and abnormal immunity have been demonstrated to play important roles in the pathogenesis of degenerative musculoskeletal diseases, such as intervertebral disc degeneration and osteoarthritis [24, 25]. Furthermore, patients with fractures suffer from severe inflammation caused by their fractures [26, 27]. Therefore, patients receiving herniorrhaphy for reducible inguinal or femoral hernia from March 2020 to March 2021 at our hospital were selected as the controls in the control group. The following patients were excluded from this study: (1) patients with trauma, immune diseases (e.g., ankylosing spondylitis, and rheumatoid arthritis), infection, or tumors affecting the spine; (2) patients with the ossification of the posterior longitudinal ligament or thoracic disc herniation; (3) patients with incarcerated hernia or strangulated hernia; and (4) patients lacking the clinical variables of interest. The diagnosis of thoracic OLF was based on the radiographic findings in thoracic computerized tomography (Figure 1). Finally, 95 patients (48 in the OLF group and 47 in the control group) were included in this retrospective study.

### 2.2. Collection of Demographic and Clinical Variables

**Figure 2:**
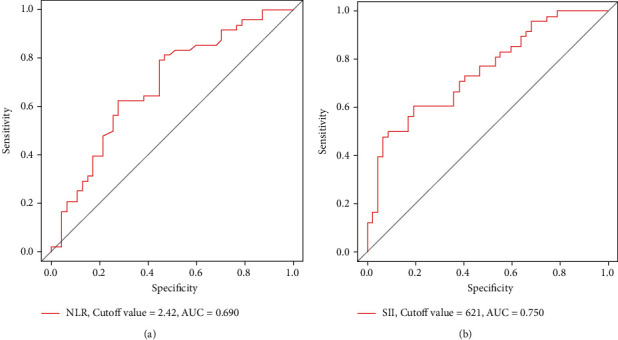
Determination of cut-off values. (a) NLR. (b) SII.

The following variables were collected for each included patient: gender; age; BMI; coexistence of hypertension or diabetes; and preoperative peripheral blood cells, including neutrophils, platelets, monocytes, and lymphocytes. The indexes of interest were calculated as follows: NLR = neutrophil counts/lymphocyte counts, PLR = platelet counts/lymphocyte counts, MLR = monocyte count/lymphocyte counts, and SII = platelet counts × neutrophil counts/lymphocyte counts. The OLF score of patients with thoracic OLF referred to the sum of segments affected by the ossified ligamentum flavum as previously reported [28]. The cut-off values of NLR and SII were set as 2.42 (Figure 2(a)) and 621 (Figure 2(b)), respectively, on the basis of the ROC curve. The cut-off value of age was set as 60 years old to distinguish between the young and elderly. The cut-off value of BMI was set as 25 kg/m^2^ to distinguish between overweight patients and patients with normal weight.

### 2.3. Statistical Analysis

All analyses in this study were conducted by using the SPSS 22.0 software package (SPSS, IBM, Chicago, IL, USA). Continuous variables, including age, BMI, NLR, PLR, MLR, and SII, were compared between two groups by using the independent samples *t*-test. Dichotomous variables, including gender and the coexistence of hypertension or diabetes, were compared between two groups by using the *χ*^2^ test. Variables with *p* < 0.10 in univariate analysis were included in the multivariate logistic regression analysis for further analysis. Variables with *p* < 0.05 in multivariate logistic regression analysis were considered as the independent risk factors of thoracic OLF and shown in the form of odds ratio (OR) with the corresponding 95% confidence interval (CI). The correlation between OLF score and BMI or SII was analyzed by using Spearman's correlation test. Here, *p* < 0.05 indicated a statistically significant result.

## 3. Results

### 3.1. General Information

The demographics of the included patients are listed in Table 1. Ninety-five patients consisting of 56 males and 39 females were included in this study. The mean age and BMI of the included patients were 51.78 ± 16.18 years and 25.31 ± 5.48 kg/m^2^, respectively. Moreover, 24 patients had coexisting hypertension or diabetes. The OLF and control groups comprised 48 and 47 patients, respectively.

### 3.2. Univariate Analysis for Potential Risk Factors

As listed in Table 2, 95 patients (56 males and 39 females) were finally included in this research. In terms of demographic variables, the OLF group had a significantly higher age (55.23 years versus 48.19 years, *p* = 0.03) and BMI (27.67 versus 22.89, *p* < 0.01) than the control group. For inflammation-related variables, the OLF group had an obviously higher NLR (2.66 versus 2.16, *p* < 0.01) and SII (636.14 versus 435.25, *p* < 0.01) than the control group. The two groups showed no significant differences in terms of gender (*p* = 0.26), the coexistence of hypertension or diabetes (*p* = 0.38), PLR (*p* = 0.39), or MLR (*p* = 0.11).

### 3.3. Multivariate Logistic Regression Analysis for Independent Risk Factors

Variables with *p* < 0.10 in univariate analysis were further included in multivariate logistic regression analysis to determine the independent risk factors of thoracic OLF. As listed in Table 3, high BMI (≥25 kg/m^2^) (OR = 9.17, 95%CI = 3.22–26.08, *p* < 0.01) and SII (≥621) (OR = 12.16, 95%CI = 2.95–50.17, *p* < 0.01) were independent risk factors of thoracic OLF. However, age (*p* = 0.20) and NLR (*p* = 0.48) were not significantly related to thoracic OLF after adjusting for all variables.

**Figure 3:**
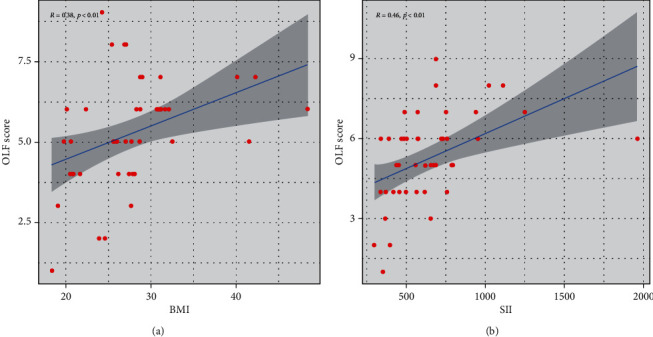
Spearman correlation analysis. (a) BMI and OLF score. (b) SII and OLF score.

Correlation analysis between OLF score and BMI or SII was performed to determine the relationship of SII and BMI with OLF further. As shown in Figure 3, BMI (*R* = 0.38, *p* < 0.01) (Figure 3(a)) and SII (*R* = 0.46, *p* < 0.01) (Figure 3(b)) were significantly and positively related to OLF score.

## 4. Discussion

OLF has become the leading cause of thoracic spinal stenosis [1, 2]. Inflammation has been proven to play important roles in the pathogenesis of OLF, and the development of OLF is associated with several inflammatory cytokines, such as IL-6 and TNF-*α* [12, 29]. However, these inflammatory cytokines are rarely examined in clinical practice. Clinical models based on peripheral blood cells have been validated to have good clinical application in human diseases [17, 22, 23]. In the current study, we discovered that high SII (≥621) and BMI (≥25 kg/m^2^) are independent risk factors of OLF. To the best of our knowledge, we are the first to demonstrate that high SII (≥621) is an independent risk factor of OLF.

SII was introduced as an indicator of the systemic inflammatory status in the body and has attracted increasing attention from researchers [30]. SII can serve as a valuable index for predicting the clinical outcomes of human diseases, especially cancers [31–33]. High SII is associated with the poor prognosis of several types of cancer, such as bladder cancer [31], cervical cancer [34], oral cancer [35], gastric cancer [36], and breast cancer [36]. In the current study, for the first time, we found that high SII is an independent risk factor of OLF and that SII is positively associated with OLF score. The underlying mechanism of inflammation in the pathogenesis of OLF has not been fully investigated. Inflammatory cells have been proven to execute important functions in ectopic ossification [37–44]. For example, Herath et al. showed that neutrophils could significantly promote the osteogenesis of osteoblasts [37] and found that autologous neutrophils could facilitate new bone formation in rabbits with calvarial bone defects [38]. Zhang and Wang reported that in tendons subjected to repetitive mechanical loading, the increase in PGE2 might induce the ossification of tendon tissues [39]. They also considered that neutrophils participated in ectopic ossification because a large proportion of PGE2 originated from neutrophils [40]. Xia et al. discovered that platelet lysate could improve the osteogenic capability of bone marrow-derived mesenchymal stem cells [41]. Karakayali et al. reported that platelet-rich plasma could promote regenerated bone consolidation and increase bone mineral density during distraction osteogenesis [42]. Ranganathan et al. found that the development of ectopic ossification was attenuated in mice deficient in B- and T-lymphocytes [43]. Kanai and Kakiuchi observed that the response levels of peripheral lymphocytes to the anti-CD3 monoclonal antibody differed between the continuous-type and the segmental-type ossification of the posterior longitudinal ligament; this difference indicated that lymphocytes might play important roles in ectopic ossification [44]. SII is calculated on the basis of the counts of platelets, neutrophils, and lymphocytes. Although researchers have attempted to explore the role of inflammatory cells in osteogenesis [37–44], how neutrophils, platelets, and lymphocytes promote the development of OLF remains unclear. Future studies should further explore the role of neutrophils, platelets, and lymphocytes in the pathogenesis of OLF.

High BMI (≥25 kg/m^2^) is another independent risk factor of OLF. Obesity or overweight is involved in the development of several human diseases, including OLF [9, 45–47]. Chang et al. reported that patients with thoracic OLF had a higher BMI than those without thoracic OLF (*p* < 0.01) [45]. Similarly, Endo et al. observed a higher BMI in the thoracic OLF group than in the control group (*p* < 0.01) and detected a significantly positive relationship between BMI and OLF score [46]. Moreover, Tang et al. reported that new OLF was usually observed in patients with high BMI; notably, reduced BMI was observed in patients with OLF disappearance within the 5-year follow-up (*p* < 0.01) [9]. In this study, we also discovered that BMI is associated with the development of OLF, and the subsequent multivariate logistic regression analysis revealed that BMI is an independent risk factor of OLF. Moreover, we observed a significantly positive relationship between BMI and OLF score. Although numerous studies have identified the unfavorable role of high BMI in the pathogenesis of OLF, the underlying mechanism of this role remains unclear. The leptin secreted by adipose tissue might be a potential reason for the tendency of patients with high BMI to develop OLF. An increased level of leptin was observed in patients with OLF, and leptin could promote ligamentum flavum cells via the activation of STAT3, JNK, and ERK1/2 [47]. Future studies should focus on the mechanistic investigation of the role of high BMI in the pathogenesis of OLF.

Some limitations should be considered when interpreting our findings. First, this study had a retrospective design and small sample size that might reduce the accuracy of its results. Second, we selected patients with reducible hernia as the control individuals. However, patients with reducible hernia are not same as the healthy population. This situation might affect the generalizability of our findings. Third, inflammatory cells in peripheral blood are easily influenced by the external environment, such as bacterial or viral infections. Fourth, blood samples were collected preoperatively, and the change in SII during the pathogenesis of OLF was not confirmed given the retrospective design of this study. Fifth, some of the included patients were diagnosed with hypertension or diabetes, which might affect the SII results [48, 49]. Multicenter prospective studies with strict methodologies and large populations should be conducted to further determine the relationship between SII and OLF and explore its underlying mechanism.

## 5. Conclusion

High SII and BMI are independent risk factors of OLF. SII and BMI are positively associated with OLF score. Inflammation and obesity might contribute to the pathogenesis of OLF.

## Figures and Tables

**Table 1:** Demographics of included patients.

Variables	Patients (*n* = 95)
Gender (*n*/%)	
Male	56 (59%)
Female	39 (41%)
Age (mean ± SD), (year)	51.78 ± 16.18
BMI (mean ± SD), (kg/m^2^)	25.31 ± 5.48
Coexistence of hypertension or diabetes (*n*/%)	
No	71 (75%)
Yes	24 (25%)
Groups	
OLF patients	48 (51%)
Control patients	47 (49%)

SD: standard deviation; BMI: body mass index.

**Table 2:** Univariate analysis for potential risk factors of thoracic OLF.

Variables	OLF group (*n* = 48)	Control group (*n* = 47)	*p*
Gender			0.26
Male	31/65%	25/53%	
Female	17/35%	22/47%	
Age (year)	55.23 ± 10.67	48.19 ± 10.82	0.03^∗^
BMI (kg/m^2^)	27.67 ± 6.05	22.89 ± 3.47	<0.01^∗^
Coexistence of hypertension or diabetes			0.38
No	34/70%	37/79%	
Yes	14/30%	10/21%	
NLR	2.66 ± 0.88	2.16 ± 0.87	<0.01^∗^
PLR	134.35 ± 46.56	142.45 ± 44.18	0.39
MLR	0.21 ± 0.07	0.24 ± 0.10	0.11
SII	636.14 ± 288.16	435.25 ± 150.12	<0.01^∗^

OLF: ossification of the ligamentum flavum; BMI: body mass index; NLR: neutrophil-to-lymphocyte ratio; PLR: platelet-to-lymphocyte ratio; MLR: monocyte-to-lymphocyte ratio; SII: systemic immune inflammation index. ^∗^Variables with *p* < 0.10 were further included into the multivariate logistic regression analysis.

**Table 3:** Multivariate analysis for independent risk factors of thoracic OLF.

Variables	OR (95% CI)	*p*
Age (year) (≥60 versus <60)	2.14 (0.67-6.85)	0.20
BMI (kg/m^2^) (≥25 versus <25)	9.17 (3.22-26.08)	<0.01^∗^
NLR (≥2.42 versus <2.42)	1.58 (0.45-5.58)	0.48
SII (≥621 versus <621)	12.16 (2.95-50.17)	<0.01^∗^

OLF: ossification of the ligamentum flavum; OR: odds ratio; CI: confidence interval; BMI: body mass index; NLR: neutrophil-to-lymphocyte ratio; PLR: platelet-to-lymphocyte ratio; MLR: monocyte-to-lymphocyte ratio; SII: systemic immune inflammation index. ^∗^Variables with *p* < 0.05 were considered as independent risk factors.

## Data Availability

The original contributions presented in the study are included in the article, and further inquiries can be directed to the corresponding author/s.

## Abbreviations

OLF: Ossification of the ligamentum flavum
BMI: Body mass index
NLR: Neutrophil-to-lymphocyte ratio
PLR: Platelet-to-lymphocyte ratio
MLR: Monocyte-to-lymphocyte ratio
SII: Systemic immune-inflammation index.
